# Assessment of risk scores to predict mortality of COVID-19 patients admitted to the intensive care unit

**DOI:** 10.3389/fmed.2023.1130218

**Published:** 2023-04-20

**Authors:** Matheus Carvalho Alves Nogueira, Vandack Nobre, Magda Carvalho Pires, Lucas Emanuel Ferreira Ramos, Yara Cristina Neves Marques Barbosa Ribeiro, Rubia Laura Oliveira Aguiar, Flavia Maria Borges Vigil, Virginia Mara Reis Gomes, Camila de Oliveira Santos, Davi Mesquita Miranda, Pamela Andrea Alves Durães, Josiane Moreira da Costa, Alexandre Vargas Schwarzbold, Angélica Gomides dos Reis Gomes, Bruno Porto Pessoa, Carolina Cunha Matos, Christiane Corrêa Rodrigues Cimini, Cíntia Alcântara de Carvalho, Daniela Ponce, Euler Roberto Fernandes Manenti, Evelin Paola de Almeida Cenci, Fernando Anschau, Flávia Carvalho Cardoso Costa, Francine Janaina Magalhães Nascimento, Frederico Bartolazzi, Genna Maira Santos Grizende, Heloisa Reniers Vianna, Jomar Cristeli Nepomuceno, Karen Brasil Ruschel, Liege Barella Zandoná, Luís César de Castro, Maíra Dias Souza, Marcelo Carneiro, Maria Aparecida Camargos Bicalho, Mariana do Nascimento Vilaça, Naiara Patrícia Fagundes Bonardi, Neimy Ramos de Oliveira, Raquel Lutkmeier, Saionara Cristina Francisco, Silvia Ferreira Araújo, Polianna Delfino-Pereira, Milena Soriano Marcolino

**Affiliations:** ^1^Department of Internal Medicine, Medical School and University Hospital, Universidade Federal de Minas Gerais, Belo Horizonte, Brazil; ^2^Department of Statistics, Universidade Federal de Minas Gerais, Belo Horizonte, Brazil; ^3^Hospital Metropolitano Doutor Célio de Castro, Belo Horizonte, Brazil; ^4^Centro Universitário de Belo Horizonte (UniBH), Belo Horizonte, Brazil; ^5^Faculdade de Ciências Médicas de Minas Gerais, Belo Horizonte, Brazil; ^6^Pontifícia Universidade Católica de Minas Gerais, Betim, Brazil; ^7^Hospital Risoleta Tolentino Neves, Belo Horizonte, Brazil; ^8^Universidade Federal dos Vales do Jequitinhonha e Mucuri, Diamantina, Brazil; ^9^Hospital Universitário de Santa Maria/EBSERH, Santa Maria, Brazil; ^10^Department of Internal Medicine, Universidade Federal de Santa Maria, Santa Maria, Brazil; ^11^Hospitais da Rede Mater Dei, Belo Horizonte, Brazil; ^12^Hospital Julia Kubitschek, Belo Horizonte, Brazil; ^13^Hospital Santa Rosália, Teófilo Otoni, Brazil; ^14^Hospital João XXIII, Belo Horizonte, Brazil; ^15^Faculdade de Medicina de Botucatu, Universidade Estadual Paulista Júlio de Mesquita Filho, Botucatu, Brazil; ^16^Hospital Mãe de Deus, Porto Alegre, Brazil; ^17^Hospital Universitário de Canoas, Canoas, Brazil; ^18^Hospital Nossa Senhora da Conceição and Hospital Cristo Redentor, Porto Alegre, Brazil; ^19^Instituto Orizonti, Belo Horizonte, Brazil; ^20^Hospital Santo Antônio, Curvelo, Brazil; ^21^Hospital Santa Casa de Misericórdia de Belo Horizonte, Belo Horizonte, Brazil; ^22^Hospital Universitário Ciências Médicas, Belo Horizonte, Brazil; ^23^Institute for Health Technology Assessment (IATS), Porto Alegre, Brazil; ^24^Hospital Bruno Born, Lajeado, Brazil; ^25^Hospital Metropolitano Odilon Behrens, Belo Horizonte, Brazil; ^26^Hospital Santa Cruz, Santa Cruz do Sul, Brazil; ^27^Universidade Federal de São João Del Rei, Divinópolis, Brazil; ^28^Hospital Eduardo de Menezes, Belo Horizonte, Brazil; ^29^Hospital Semper, Belo Horizonte, Brazil; ^30^Telehealth Center, University Hospital, Universidade Federal de Minas Gerais, Belo Horizonte, Brazil

**Keywords:** SARS-CoV-2, COVID-19, intensive care unit, prognosis, mortality, risk scores

## Abstract

**Objectives:**

To assess the ABC_2_-SPH score in predicting COVID-19 in-hospital mortality, during intensive care unit (ICU) admission, and to compare its performance with other scores (SOFA, SAPS-3, NEWS2, 4C Mortality Score, SOARS, CURB-65, modified CHA2DS2-VASc, and a novel severity score).

**Materials and methods:**

Consecutive patients (≥ 18 years) with laboratory-confirmed COVID-19 admitted to ICUs of 25 hospitals, located in 17 Brazilian cities, from October 2020 to March 2022, were included. Overall performance of the scores was evaluated using the Brier score. ABC_2_-SPH was used as the reference score, and comparisons between ABC_2_-SPH and the other scores were performed by using the Bonferroni method of correction. The primary outcome was in-hospital mortality.

**Results:**

ABC_2_-SPH had an area under the curve of 0.716 (95% CI 0.693–0.738), significantly higher than CURB-65, SOFA, NEWS2, SOARS, and modified CHA2DS2-VASc scores. There was no statistically significant difference between ABC_2_-SPH and SAPS-3, 4C Mortality Score, and the novel severity score.

**Conclusion:**

ABC_2_-SPH was superior to other risk scores, but it still did not demonstrate an excellent predictive ability for mortality in critically ill COVID-19 patients. Our results indicate the need to develop a new score, for this subset of patients.

## Introduction

Since its breakthrough, the COVID-19 pandemic caused a collapse of healthcare systems around the world, with an exceeding demand for intensive care beds and mechanical ventilators (1, 2). Increasing cases and widespread dissemination of SARS-CoV-2 created the perfect scenario for the acquisition of advantageous mutations, modifying viral transmissibility and disease severity, and allowing escape from natural or vaccine-mediated immunity (3, 4).

In this context, a rapid, objective, and reliable evaluation of critically ill patients is fundamental for efficient triage, as well as for treatment, and resource allocation. Patients with COVID-19 may deteriorate rapidly after a period of reasonably mild symptoms, reinforcing the need for early risk stratification (5, 6).

Our research group has developed the ABC_2_-SPH score, which is the only score developed and validated in Brazilian COVID-19 patients. It uses strict methodological criteria, with few, easily obtained clinical and laboratory data at hospital presentation to predict in-hospital mortality. ABC_2_-SPH score has shown high accuracy to discriminate between high-risk and non-high-risk patients, superior to several other scores in a large sample of Brazilian patients (7). Nevertheless, this score has not been validated yet to be applied at ICU admission.

Therefore, our aim was to assess the ABC_2_-SPH score, during intensive care unit (ICU) admission, in predicting COVID-19 in-hospital mortality, and to compare its performance with other scores: Sequential Organ Failure Assessment (SOFA), Simplified Acute Physiology Score III (SAPS-3), National Early Warning Score 2 (NEWS2), 4C Mortality Score, SOARS, CURB-65, modified CHA2DS2-VASc, and a novel severity score.

## Materials and methods

This study is part of the Brazilian COVID-19 Registry, a retrospective multicenter cohort, which included data from 25 hospitals in Brazil, in 17 cities, with a total of 752 ICU beds, described in detail elsewhere (7).

### Study subjects

**Figure 1:**
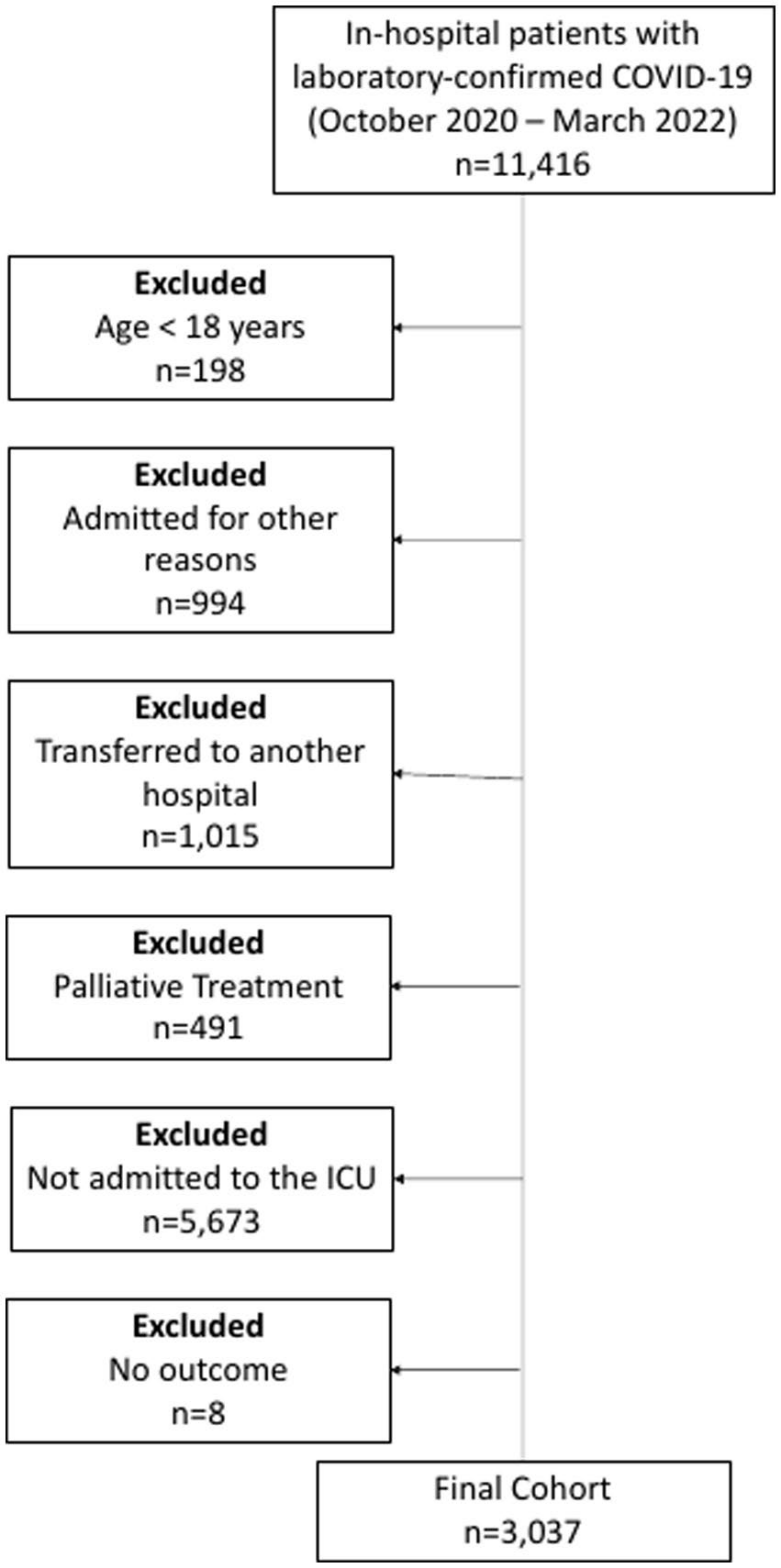
Flowchart of COVID-19 patients included in the study.

Consecutive patients (aged ≥18 years) with laboratory-confirmed COVID-19 (positive SARS-CoV-2 RT-PCR or rapid antigen test), according to World Health Organization guidance, admitted to the ICUs of one of the participating hospitals, between 4 October 2020, and 13 March 2022, were included. Patients with missing data in any of the variables used for the ABC_2_-SPH score, as well as pregnant patients and those who were admitted for other reasons and developed COVID-19 during their hospital stay were not included in this analysis (Figure 1).

### Data collection

Demographic information, clinical characteristics, laboratory findings, therapeutic interventions, and outcomes were collected by trained researchers from patient charts to the Research Electronic Data Capture (REDCap) electronic platform, hosted at the Telehealth Center of the *Hospital das Clínicas of Universidade Federal de Minas Gerais (UFMG)* (8–10). For analysis, only the first ICU admission was considered if the patient had two distinct admissions in the same hospital stay.

Periodical data quality checks were performed to ensure data accuracy. Values likely related to data entry errors were identified using a code developed in R software, based on expert-guided rules. Those data were sent to each center for checking and correction (7).

### Sample size

Standardized methodology from the Transparent Reporting of a Multivariable Prediction Model for Individual Prediction or Diagnosis (TRIPOD) checklist (11) recommends that ideally, at least 250 events (in this case, deaths) and 250 non-events should be included for score validation. In the present analysis, there was no formal sample size calculation. Instead, all eligible patients were included, with a sample size that met those requirements.

### ABC_2_-SPH

The ABC_2_-SPH score was developed, validated, and reported following guidance from the TRIPOD checklist (11, 12) and the Prediction model Risk Of Bias Assessment Tool (PROBAST) (13).

The score was derived from a population of 3,978 hospital inpatients, from 36 hospitals, using data upon hospital presentation. Validation was conducted on 1,054 inpatient records from the same institutions (temporal validation) and also on patients from the Vall d’Hebron University Hospital cohort (external validation) (7, 14).

The score incorporates the following variables: **A**ge, **B**UN (blood urea nitrogen), **C**omorbidities, **C**-reactive protein, **S**pO_2_/FiO_2_ ratio, **P**latelet count, and **H**eart rate. The score ranges from 0 to 20, with risk groups defined as low (0–1), intermediate (2–4), high (5–8), and very high (≥ 9). In the validation cohorts, it has shown high discriminatory ability, with AUROC of 0.859 (95% CI 0.833–0.885) and 0.894 (95% CI 0.870–0.919) for the Brazilian and Spanish cohorts, respectively, and displayed better discrimination ability than other existing scores (7).

### Comparison with other risk scores

The accuracy of the ABC_2_-SPH score was compared with that of other scores developed specifically for COVID-19. Additionally, we compared the ABC_2_-SPH score with scores developed for other conditions, such as pneumonia and sepsis, applied in severely ill or ICU patients and with early warning scores. The scores used for such comparisons were chosen based on two conditions: (1) they had already been evaluated for COVID-19 in other studies, and (2) they used parameters that were available within our database, with accessible methods for calculation (described in a previous publication). They are SOFA (15), SAPS-3 (16, 17), NEWS2 (18), 4C Mortality Score (19, 20), SOARS (21), CURB-65 (22), and a novel severity score developed by Altschul et al. (23). A modified version of the CHA2DS2-VASc score tested in a previous publication to assess mortality in ICU COVID-19 patients (scoring for male sex instead of female) was included in the comparison as well (24). Model comparisons were performed using AUROC and the decision curve analysis.

### Outcome

The primary outcome was all-cause in-hospital mortality (considering the entire period of hospitalization).

### Statistical analysis

Continuous variables were summarized as medians and interquartile ranges (IQR), and categorical variables as counts and percentages. Data were imputed for variables with up to 30% missing values. This study reported 95% confidence intervals (CI), and a *p*-value < 0.05 was considered statistically significant. Statistical analysis was performed using the free software R (version 4.0.2), and the packages tidyverse, gt, gtsummary, ggplot2, and rms (25).

ABC_2_-SPH was used as the reference score for every comparison since it is the only mortality risk score for COVID-19 tested and validated in the Brazilian population (7). Comparisons between ABC_2_-SPH and the other scores were performed by the Bonferroni correction method.

### Performance measures

The area under the receiver operating characteristic curve (AUROC) described the models’ discrimination Confidence intervals for AUROC were obtained across 2,000 bootstrap samples.

Overall performance of the scores was evaluated using the Brier score (26). Only the ABC_2_-SPH, SAPS-3, and 4C Mortality scores provided data that allowed calibration. It was performed by plotting the predicted mortality probabilities against the observed mortality, testing intercept equals zero and slope equals one.

We further performed a subgroup analysis comprising the worst phase of the pandemic in Brazil (between 1 March 2021, and 30 April 2021), according to epidemiological data provided by the Brazilian Ministry of Health (27).

## Results

A total of 3,037 patients were included, 55.9% were men, with a median age of 61 (IQR 50–70) years old and overall mortality of 50.0%. When comparing patients who died with those who were discharged alive from the hospital, the first group was older and had a higher prevalence of underlying comorbidities such as hypertension, coronary artery disease, heart failure, chronic obstructive pulmonary disease, and cancer, moreover lower platelet levels, higher urea, and C-reactive protein levels, at ICU admission (Supplementary Table S1).

**Figure 2:**
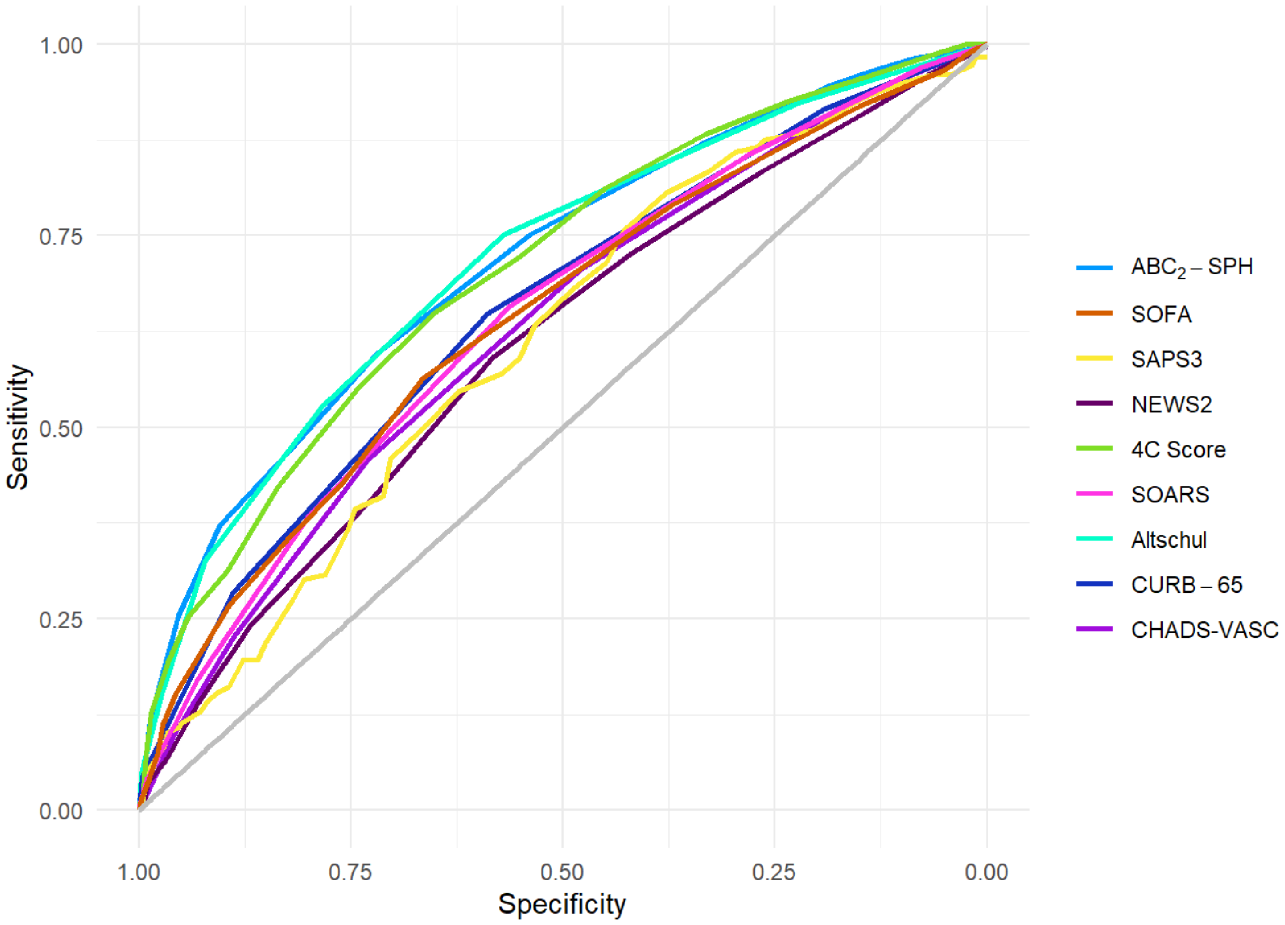
Discrimination of ABC_2_-SPH and other scores in this cohort.

Table 1 and Figure 2 show the discrimination ability expressed as the AUROC for each of the scores evaluated, while Table 2 depicts the results of the statistical comparison between these scores and ABC_2_-SPH, selected as the reference score.

**Table 1:** Discrimination ability for each score applied in the database of COVID-19 patients admitted to the intensive care unit.

Model	N*	AUROC (95%CI)
ABC_2_-SPH	1,823	0.716 (0.693–0.738)
Altschul et al. (23)	1,334	0.715 (0.688–0.742)
4C Mortality Score	985	0.706 (0.673–0.739)
CURB-65	2,149	0.652 (0.630–0.675)
SOARS	2,515	0.642 (0.621–0.662)
SOFA	928	0.642 (0.601–0.678)
Modified CHA2DS2-VASc	2,787	0.628 (0.608–0.648)
SAPS-3	541	0.614 (0.566–0.663)
NEWS2	1,095	0.605 (0.574–0.637)

*Complete case analysis. Data were imputed for variables with up to 30% missing values.

**Table 2:** Comparison between ABC_2_-SPH and other scores.

Reference score	Compared score	*p*-value	alpha^*^	*N*	Result
ABC_2_-SPH	Altschul et al. (23)	0.9346	0.0063	1,094	AUROC of ABC_2_-SPH is not different
ABC_2_-SPH	4C Mortality Score	0.8878	0.0063	815	AUROC of ABC_2_-SPH is not different
ABC_2_-SPH	CURB-65	**0.0010**	0.0063	1,823	AUROC of ABC_2_-SPH is larger^**^
ABC_2_-SPH	SOARS	**0.0000**	0.0063	2,147	AUROC of ABC_2_-SPH is larger^**^
ABC_2_-SPH	SOFA	**0.0032**	0.0063	842	AUROC of ABC_2_-SPH is larger^**^
ABC_2_-SPH	Modified CHA2DS2-VASC	**0.0000**	0.0063	2,380	AUROC of ABC_2_-SPH is larger^**^
ABC_2_-SPH	SAPS-3	0.0446	0.0063	539	AUROC of ABC_2_-SPH is not different
ABC_2_-SPH	NEWS2	**0.0000**	0.0063	976	AUROC of ABC_2_-SPH is larger^**^

AUROC, area under the ROC curve. ^*^Due to the multiple comparisons, alpha was corrected using Bonferroni method. ^**^ABC_2_-SPH has higher discrimination ability.

As seen in Table 2, ABC_2_-SPH had higher discrimination than CURB65, SOFA, NEWS2, SOARS, and modified CHA2DS2-VASc scores (AUROC: 0.716 [95% CI 0.693–0.738]). There was no statistically significant difference between ABC_2_-SPH and SAPS-3, 4C Score, and the novel score by Altschul. Even though the AUROC of SAPS-3 was the second lowest in absolute terms (0.614, 95% CI 0.566–0.663), there was no statistically significant difference between that and the ABC_2_-SPH score. SAPS-3 had the smallest sample, with only 541 patients included in the analysis, and this might explain the lack of significance.

**Figure 3:**
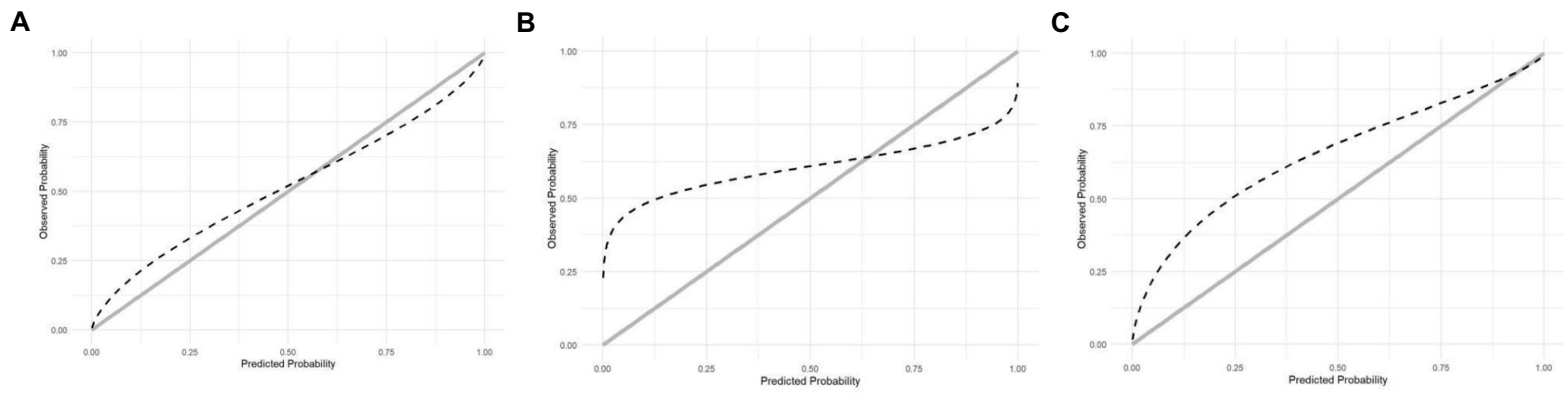
**(A)** Calibration of ABC_2_-SPH. **(B)** Calibration of SAPS3. **(C)** Calibration of 4C Score.

The calibration curve indicates that the ABC_2_-SPH underestimated mortality at lower ranges of the score and overestimated it at the higher ones. In other words, the less severely ill patients have had a worse outcome than the score could predict, as seen in Figure 3A. SAPS-3 had an even greater underestimation of mortality at lower ranges and overestimation at the higher ranges (Figure 3B). The 4C Mortality score, on the other hand, underestimated mortality through all the ranges of the score (Figure 3C). The calibration curves could not be produced for the remaining scores because it was not possible to access their original derivation data.

## Discussion

In the present study, ABC_2_-SPH presented a reasonable performance when applied during ICU admission in predicting COVID-19 in-hospital mortality, and it was significantly better than CURB-65, SOFA, NEWS2, SOARS, and the modified version of CHA2DS2-VASc. When comparing the performance of the ABC_2_-SPH to the SAPS-3, 4C Score, and the score by Altschul et al., we did not observe significant differences.

In the context of the COVID-19 pandemic, many new risk scores were developed and others were tested, or even adapted. A modified version of CHA2DS2-VASc score (giving 1 point for the male sex and 0 points for the female sex, considering male sex a risk factor for COVID-19) was evaluated in 209 intensive care patients, with the rationale that endothelial dysfunction and thrombosis are important components of COVID-19 pathophysiology, but it had fair results (24).

Most of the studies carried out to test or develop risk scores for COVID-19 patients at ICU admission used small samples, increasing the imprecision and compromising the external validity of the results. For instance, a prospective study compared different early warning scores, applied at admission to the ICU, to predict mortality in 140 critically ill patients with laboratory-confirmed COVID-19 (18). The overall performance was intermediate, and the confidence intervals were too wide, conferring significant imprecision to the results. CRB-65, the best discriminatory tool in that study, showed an AUC of 0.720 (95% CI 0.630–0.811).

In a larger study, the performance of SAPS-3 was evaluated in 30,571 COVID-19 patients admitted to ICUs in Brazil. The model’s discrimination was excellent, with an AUROC of 0.835 (95% CI 0.828–0.841). However, the mortality was considerably lower than in our cohort (15.0% vs. 50.0%), as well as in other studies with critically ill COVID-19 patients from varied countries, which had a mortality rate between 26 to 50% (28–33). The low mortality rate may have influenced SAPS-3 outperformance in that specific study. Still, the calibration was inappropriate, with an underestimation of mortality in lower to intermediate-risk groups, and an overestimation in the higher-risk group (16).

An Italian group developed and internally validated a prediction model for 28-day mortality of critically ill COVID-19 patients admitted to the ICU. This study used clinical variables (age, obesity, procalcitonin, SOFA score, and PaO_2_/FiO_2_ ratio), with an excellent discriminatory capacity of 0.821 (95% CI 0.766–0.876) and 0.822 (95% CI 0.770–0.873), in the original and bootstrap models, respectively (34). Nevertheless, some limitations should be mentioned: the model lacks external validation, the authors included a relatively small sample of participants, and the inclusion of serum procalcitonin (a less available laboratory test) limits the widespread use of this score.

In a multicenter cohort in Italy, a machine learning (ML) approach was applied for the development and validation of a predictive model, utilizing many clinical variables. The performance was better when the variables were collected both at ICU admission and during ICU stay (even though with more than 85% of missing data) and were less satisfactory considering only the variables collected at ICU admission that had less than 85% of missing data (35). The sample was modest for a ML approach, with only 1,293 patients for score development, and less than 100 events in the external validation datasets. Still, there was no information on the imprecision of the results, as the authors did not provide the confidence intervals.

Knight et al. (2020) developed and validated the 4C Mortality Score, which uses eight variables readily available at hospital admission, with reasonable discrimination for mortality (AUC 0.774, CI 95% 0.767–0.782) and excellent calibration. Nevertheless, this score was aimed to be used at the moment of hospital admission, not necessarily at ICU admission, and has not been validated for such use (20).

A multicenter retrospective cohort study carried out in Spain and conducted on patients transferred by ambulance to an emergency department evaluated the NEWS2 performance. The NEWS2 score provided an AUROC ranging from 0.825 for 1-day mortality to 0.777 for 90-day mortality. Nevertheless, the hospitalization rate of the 2,961 patients included was 78.6%, while patients that required ICU admission represented only 5.5% of the total participants, and no subgroup analysis was made (36).

The validation of the ABC_2_-SPH in a large cohort of patients admitted to ICU due to COVID-19 complications could be helpful, given that other scores proved to be inaccurate in this scenario. Nevertheless, despite its excellent discrimination for mortality at hospital admission, the results were only reasonable when applied at ICU admission. The AUROC of 0.716 (95% CI 0.693–0.738) was considerably inferior to that observed in the original study (7). The same happened with the widely used SAPS-3, SOFA, and NEWS2, as described above, which had a worse performance than ABC_2_-SPH.

We initially hypothesized that one of the reasons that could explain such unsatisfactory performances is that our cohort was composed exclusively of patients from Brazilian hospitals, including patients admitted during the worst wave of the pandemic in Brazil (27). This could have affected the performance of the scores, since the collapse of the health system may have led many patients to be admitted to ICUs at late phases of the disease, making their recovery more difficult. Another possibility could be that, under the huge saturations of the ICUs during the worst waves, the most critically ill patients did not get admitted into the ICU, with the ones with a better prognosis getting the priority. Nevertheless, in a subgroup analysis of the patients evaluated during the worst phase of the pandemic in Brazil, between 1 March 2021 and 30 April 2021, there was no significant difference in the performance of ABC_2_-SPH (Supplementary Table S2).

Some aspects of each score may have had a negative impact on their performance in this study. ABC_2_-SPH, for instance, uses the SF ratio (SpO_2_/FiO_2_) as one of its parameters: the lower the ratio, the higher the score, indicating a higher probability of death. Nevertheless, patients admitted to the ICU are frequently on mechanical ventilation (38.1% of all patients evaluated, being 49.1% among those who died and 27.3% among the survivors), which may lead to an inadequate degree of hyperoxia, not necessarily a less severe clinical state, and this could potentially mislead the score.

Besides that, of all the parameters included in ABC_2_-SPH, involving different organ systems, only the SF ratio is directly related to the respiratory system, which is the main cause of death in COVID-19 patients (37). Perhaps, the inclusion of more parameters related to the respiratory system, such as the severity of lung involvement in computerized tomography, could improve the accuracy of the score. The use of imaging methods might cause some mistrust, being it operator-dependent, but the development of machine-learning techniques could eventually surpass this issue.

On the other hand, SOFA includes the mean arterial pressure as one of its parameters, giving it the same value as PaO_2_/FiO_2_ ratio for the score (0 to 4 points). Nevertheless, unlike respiratory impairment, hypotension does not seem to be part of the main core of COVID-19 mortality, in the absence of a specific cause.

Likewise, SAPS-3 uses many different parameters which might not be as relevant for COVID-19 mortality. Age just above 40 years already scores 5 points, enough to almost double the probability of death. In contrast, according to our database, the risk of death in the age group of 40–49 years old is 33.5%, compared to 25.6% of those aged 18–29 years old. The risk of death, in reality, only doubles in the age group of 60–69 years old (54.1%) (Supplementary Table S3). Furthermore, SAPS-3 includes a large number of variables that do not apply to our set of patients, such as the reason for ICU admission (in this study, admission for some reason other than COVID-19 was an exclusion criterion). And the same way that SOFA, mean arterial pressure is as valued as PaO_2_/FiO_2_ ratio.

Therefore, we hypothesized that such imbalances between clinical importance and the weight of each variable included in the scores could be a reason for such unsatisfactory performances.

This study has limitations that deserve comments. Hospitals from different regional settings and different sizes were included in the study to increase external validity. However, infrastructure unbalances between them may have impacted the results. In addition, some of the scores ended up with fewer participants than others due to incomplete data, since data were imputed for variables with up to 30% missing values. SAPS-3, as mentioned above, is an example of that. Furthermore, the scores chosen to be included in the analysis were limited to the parameters available within our database, leaving some others out of the study.

Further and periodical adjustments, in a similar manner that happens with other risk scores which are subjected to continuous updates (such as APACHE and SAPS), should also be considered for ABC_2_-SPH.

## Conclusion

In this study, applying ABC_2_-SPH at ICU admission had a reasonable performance in predicting in-hospital mortality of COVID-19 critically ill patients, superior to other risk scores. In order to obtain excellent performance, nevertheless, it may be necessary to develop a new score for this specific subset of patients.

## Data availability statement

The raw data supporting the conclusions of this article will be made available by the authors, without undue reservation.

## Ethics statement

The study protocol was approved by the Brazilian National Commission for Research Ethics (CAAE: 30350820.5.1001.0008) on April 2, 2020, with the following title (free translation): “Evaluation of the laboratory, radiological and symptomatological profile of infected patients with the new coronavirus 2019 (SARS-CoV-2) in hospitals in the State of Minas Gerais.” The study title has been changed on 10 June 2020 to “National multicenter hospital registry of patients with disease caused by SARS-COV-2 [COVID-19],” also in free translation. Individual informed consent was waived by a regulatory board due to the severity of the situation and the use of deidentified data, based on medical chart review only. This study followed the Strengthening the Reporting of Observational Studies in Epidemiology (STROBE) guidelines for observational cohort studies and it is in accordance with the Declaration of Helsinki.

## Author contributions

MSM, VN, MCP, LEFR, and YCNMBR: conception or design of the work. MCAN, VN, MCP, LEFR, YCNMBR, RLOA, FMBV, VMRG, COS, DMM, PAAD, JMC, AVS, AGRG, BPP, CCM, CCRC, CAC, DP, ERFM, EPAC, FA, FCCC, FJMN, FB, GMSG, HRV, JCN, KBR, LBZ, LCC, MDS, MC, MACB, MNV, NPFB, NRO, RL, SCF, SFA, PDP, and MSM: acquisition, analysis, or interpretation of data for the work. MCAN, VN, PDP, and MSM: drafted the work. MSM and MCAN agreed to be accountable for all aspects of the work in ensuring that questions related to the accuracy or integrity of any part of the work are appropriately investigated and resolved. All authors were revised the manuscript critically for important intellectual content and gave final approval of the version to be published.

## Funding

This study was supported in part by Minas Gerais State Agency for Research and Development (Fundação de Amparo à Pesquisa do Estado de Minas Gerais—FAPEMIG) [grant number APQ-01154-21], National Institute of Science and Technology for Health Technology Assessment (Instituto de Avaliação de Tecnologias em Saúde—IATS)/National Council for Scientific and Technological Development (Conselho Nacional de Desenvolvimento Científico e Tecnológico—CNPq) [grants numbers 465518/2014-1 and 147122/2021-0] and CAPES Foundation (Coordenação de Aperfeiçoamento de Pessoal de Nível Superior) [Grant Number 88887.507149/2020-00].

## Conflict of interest

The authors declare that the research was conducted in the absence of any commercial or financial relationships that could be construed as a potential conflict of interest.

## Publisher’s note

All claims expressed in this article are solely those of the authors and do not necessarily represent those of their affiliated organizations, or those of the publisher, the editors and the reviewers. Any product that may be evaluated in this article, or claim that may be made by its manufacturer, is not guaranteed or endorsed by the publisher.

## References

[1] Walker PGT Whittaker C Watson OJ Baguelin M Winskill P Hamlet A. The impact of COVID-19 and strategies for mitigation and suppression in low- and middle-income countries. Science. (2020) 369:413–22. doi: 10.1126/science.abc0035, PMID: 32532802 PMC7292504

[2] Saha S Tanmoy AM Tanni AA Goswami S Al Sium SM Saha S. New waves, new variants, old inequity: a continuing COVID-19 crisis. BMJ Glob Health. (2021) 6:e007031. doi: 10.1136/bmjgh-2021-007031 PMC8361706 34385165

[3] CDC. Variants of the virus. Ctr Dis Control Prev. (2022).

[4] Dyson L Hill EM Moore S Curran-Sebastian J Tildesley MJ Lythgoe KA. Possible future waves of SARS-CoV-2 infection generated by variants of concern with a range of characteristics. Nat Commun. (2021) 12:5730. doi: 10.1038/s41467-021-25915-7 34593807 PMC8484271

[5] Zhao Z Chen A Hou W Graham JM Li H Richman PS. Prediction model and risk scores of ICU admission and mortality in COVID-19. PLoS One. (2020) 15:e0236618. doi: 10.1371/journal.pone.0236618, PMID: 32730358 PMC7392248

[6] Sung J Choudry N Bachour R. Development and validation of a simple risk score for diagnosing COVID-19 in the emergency room. Epidemiol Infect. (2020) 148:e273. doi: 10.1017/S0950268820002769, PMID: 33183384 PMC7729168

[7] Milena S Marcolino MDM Pires MC Ramos LEF Oliveira LM Carvalho RLR. ABC2-SPH risk score for in-hospital mortality in COVID-19 patients: development, external validation and comparison with other available scores. Int J Infect Dis. (2021) 110:281–308. doi: 10.1016/j.ijid.2021.07.049 34311100 PMC8302820

[8] Harris PA Taylor R Thielke R. Research electronic data capture (REDCap)--a metadata-driven methodology and workflow process for providing translational research informatics support. J Biomed Inform. (2009) 42:377–81. doi: 10.1016/j.jbi.2008.08.010, PMID: 18929686 PMC2700030

[9] Harris PA Taylor R Minor BL Elliott V Fernandez M O’Neal L. The REDCap consortium: building an international community of software platform partners. J Biomed Inform. (2019) 95:103208. doi: 10.1016/j.jbi.2019.103208, PMID: 31078660 PMC7254481

[10] Soriano Marcolino M Minelli Figueira R Dos Santos JPA Cardoso CS Ribeiro AL Alkmim MB. The experience of a sustainable large scale Brazilian Telehealth network. Telemed J E Health. (2016) 22:899–908. doi: 10.1089/tmj.2015.0234, PMID: 27167901

[11] Collins GS Reitsma JB Altman DG Moons KGM. Transparent reporting of a multivariable prediction model for individual prognosis or diagnosis (TRIPOD): the TRIPOD statement. BMJ. (2015) 350:g7594. doi: 10.1136/bmj.g7594 25569120

[12] Moons KGM Altman DG Reitsma JB Ioannidis JPA Macaskill P Steyerberg EW. Transparent reporting of a multivariable prediction model for individual prognosis or diagnosis (TRIPOD): explanation and elaboration. Ann Intern Med. (2015) 162:W1–W73. doi: 10.7326/M14-0698, PMID: 25560730

[13] Wolff RF Moons KGM Riley RD Whiting PF Westwood M Collins GS. PROBAST: a tool to assess the risk of bias and applicability of prediction model studies. Ann Intern Med. (2019) 170:51–8. doi: 10.7326/M18-1376 30596875

[14] Berenguer J Ryan P Rodríguez-Baño J Jarrín I Carratalà J Pachón J. Characteristics and predictors of death among 4035 consecutively hospitalized patients with COVID-19 in Spain. Clin Microbiol Infect. (2020) 26:1525–36. doi: 10.1016/j.cmi.2020.07.024, PMID: 32758659 PMC7399713

[15] Vicka V Januskeviciute E Miskinyte S Ringaitiene D Serpytis M Klimasauskas A. Comparison of mortality risk evaluation tools efficacy in critically ill COVID-19 patients. BMC Infect Dis. (2021) 21:1173. doi: 10.1186/s12879-021-06866-2, PMID: 34809594 PMC8607225

[16] Kurtz P Bastos LSL Salluh JIF Bozza FA Soares M. SAPS-3 performance for hospital mortality prediction in 30,571 patients with COVID-19 admitted to ICUs in Brazil. Intensive Care Med. (2021) 47:1047–9. doi: 10.1007/s00134-021-06474-3, PMID: 34244829 PMC8270768

[17] Metnitz PGH Moreno RP Fellinger T Posch M Zajic P. Evaluation and calibration of SAPS 3 in patients with COVID-19 admitted to intensive care units. Intensive Care Med. (2021) 47:910–2. doi: 10.1007/s00134-021-06436-9, PMID: 34009450 PMC8131881

[18] Tyagi A Tyagi S Agrawal A Mohan A Garg D Salhotra R. Early warning scores at time of ICU admission to predict mortality in critically ill COVID-19 patients. Disaster Med Public Health Prep. (2021) 18:1–5. doi: 10.1017/dmp.2021.208 PMC8376854 34140066

[19] Mumtaz SA Shahzad SA Ahmed I Alodat MA Gharba M Saif ZA. External validation of 4C ISARIC mortality score in the setting of a Saudi Arabian ICU. Retrospective study. SSRN Electron J. (2021) 10:19–24. doi: 10.1101/2021.08.16.21262104 PMC8869262 35283713

[20] Knight SR Ho A Pius R Carson G Dunning J Fairfield CJ. Risk stratification of patients admitted to hospital with covid-19 using the ISARIC WHO clinical characterisation protocol: development and validation of the 4C mortality score. BMJ. (2020) 370:3339. doi: 10.1136/bmj.m3339 PMC7116472 32907855

[21] Chua F Vancheeswaran R Draper A Vaghela T Knight M Mogal R. Early prognostication of COVID-19 to guide hospitalisation versus outpatient monitoring using a point-of-test risk prediction score. Thorax. (2021) 76:696–703. doi: 10.1136/thoraxjnl-2020-216425 33692174

[22] Neto FL Marino LO Torres A Cilloniz C Marchini JFM de Alencar JCG. Community-acquired pneumonia severity assessment tools in patients hospitalized with COVID-19: a validation and clinical applicability study. Clin Microbiol Infect. (2021) 27:1037.e1–8. doi: 10.1016/j.cmi.2021.03.002 PMC8016546 33813111

[23] Altschul DJ Unda SR Benton J de la Garza Ramos R Cezayirli P Mehler M. A novel severity score to predict inpatient mortality in COVID-19 patients. Sci Rep. (2020) 10:16726. doi: 10.1038/s41598-020-73962-9, PMID: 33028914 PMC7542454

[24] Gunduz R Yildiz BS Ozdemir IH Cetin N Ozen MB Bakir EO. CHA2DS2-VASc score and modified CHA2DS2-VASc score can predict mortality and intensive care unit hospitalization in COVID-19 patients. J Thromb Thrombolysis. (2021) 52:914–24. doi: 10.1007/s11239-021-02427-1, PMID: 33730303 PMC7970772

[25] R Core Team (2020) R a language and environment for Statistical Computing. Available at: https://scirp.org/reference/referencespapers.aspx?referenceid=3064798 (Accessed November 20, 2022).

[26] Rufibach K. Use of brier score to assess binary predictions. J Clin Epidemiol. (2010) 63:938–9. doi: 10.1016/j.jclinepi.2009.11.009 20189763

[27] Coronavírus Brasil. (2022). Available at: https://covid.saude.gov.br

[28] African COVID-19 Critical Care Outcomes Study (ACCCOS) Investigators. Patient care and clinical outcomes for patients with COVID-19 infection admitted to African high-care or intensive care units (ACCCOS): a multicentre, prospective, observational cohort study. Lancet. (2021) 397:1885–94. doi: 10.1016/S0140-6736(21)00441-4, PMID: 34022988 PMC8137309

[29] Anesi GL Jablonski J Harhay MO Atkins JH Bajaj J Baston C. Characteristics, outcomes, and trends of patients with COVID-19-related critical illness at a learning health system in the United States. Ann Intern Med. (2021) 174:613–21. doi: 10.7326/M20-5327, PMID: 33460330 PMC7901669

[30] Bhatraju PK Ghassemieh BJ Nichols M Kim R Jerome KR Nalla AK. COVID-19 in critically ill patients in the Seattle region-case series. N Engl J Med. (2020) 382:2012–22. doi: 10.1056/NEJMoa2004500 32227758 PMC7143164

[31] Tan E Song J Deane AM Plummer MP. Global impact of coronavirus disease 2019 infection requiring admission to the ICU: a systematic review and meta-analysis. Chest. (2021) 159:524–36. doi: 10.1016/j.chest.2020.10.014, PMID: 33069725 PMC7557272

[32] Potere N Valeriani E Candeloro M Tana M Porreca E Abbate A. Acute complications and mortality in hospitalized patients with coronavirus disease 2019: a systematic review and meta-analysis. Crit Care. (2020) 24:389. doi: 10.1186/s13054-020-03022-1, PMID: 32616077 PMC7330272

[33] Grasselli G Zangrillo A Zanella A Antonelli M Cabrini L Castelli A. Baseline characteristics and outcomes of 1591 patients infected with SARS-CoV-2 admitted to ICUs of the Lombardy region, Italy. JAMA. (2020) 323:1574–81. doi: 10.1001/jama.2020.5394 32250385 PMC7136855

[34] Leoni MLG Lombardelli L Colombi D Bignami EG Pergolotti B Repetti F. Prediction of 28-day mortality in critically ill patients with COVID-19: development and internal validation of a clinical prediction model. PLoS One. (2021) 16:e0254550. doi: 10.1371/journal.pone.0254550, PMID: 34255793 PMC8277063

[35] Lorenzoni G Sella N Boscolo A Azzolina D Bartolotta P Pasin L. COVID-19 ICU mortality prediction: a machine learning approach using SuperLearner algorithm. J Anesthesia Analgesia Critical Care. (2021) 1:1–10. doi: 10.1186/s44158-021-00002-x PMC8413709 37386625

[36] Martín-Rodríguez F Sanz-García A Ortega GJ Delgado-Benito JF García Villena E Mazas Pérez-Oleaga C. One-on-one comparison between qCSI and NEWS scores for mortality risk assessment in patients with COVID-19. Ann Med. (2022) 54:646–54. doi: 10.1080/07853890.2022.2042590, PMID: 35193439 PMC8881067

[37] Sjoding MW Admon AJ Saha AK Kay SG Brown CA Co I. Comparing clinical features and outcomes in mechanically ventilated patients with COVID-19 and acute respiratory distress syndrome. Ann Am Thorac Soc. (2021) 18:1876–85. doi: 10.1513/AnnalsATS.202008-1076OC, PMID: 33577740 PMC8641825

