# Corrigendum: Assessment of risk scores to predict mortality of COVID-19 patients admitted to the intensive care unit

**DOI:** 10.3389/fmed.2024.1363948

**Published:** 2024-01-31

**Authors:** Matheus Carvalho Alves Nogueira, Vandack Nobre, Magda Carvalho Pires, Lucas Emanuel Ferreira Ramos, Yara Cristina Neves Marques Barbosa Ribeiro, Rubia Laura Oliveira Aguiar, Flavia Maria Borges Vigil, Virginia Mara Reis Gomes, Camila de Oliveira Santos, Davi Mesquita Miranda, Pamela Andrea Alves Durães, Josiane Moreira da Costa, Alexandre Vargas Schwarzbold, Angélica Gomides dos Reis Gomes, Bruno Porto Pessoa, Carolina Cunha Matos, Christiane Corrêa Rodrigues Cimini, Cíntia Alcântara de Carvalho, Daniela Ponce, Euler Roberto Fernandes Manenti, Evelin Paola de Almeida Cenci, Fernando Anschau, Flávia Carvalho Cardoso Costa, Francine Janaina Magalhães Nascimento, Frederico Bartolazzi, Genna Maira Santos Grizende, Heloisa Reniers Vianna, Jomar Cristeli Nepomuceno, Karen Brasil Ruschel, Liege Barella Zandoná, Luís César de Castro, Maíra Dias Souza, Marcelo Carneiro, Maria Aparecida Camargos Bicalho, Mariana do Nascimento Vilaça, Naiara Patrícia Fagundes Bonardi, Neimy Ramos de Oliveira, Raquel Lutkmeier, Saionara Cristina Francisco, Silvia Ferreira Araújo, Polianna Delfino-Pereira, Milena Soriano Marcolino

**Affiliations:** ^1^Department of Internal Medicine, Medical School and University Hospital, Universidade Federal de Minas Gerais, Belo Horizonte, Brazil; ^2^Department of Statistics, Universidade Federal de Minas Gerais, Belo Horizonte, Brazil; ^3^Hospital Metropolitano Doutor Célio de Castro, Belo Horizonte, Brazil; ^4^Centro Universitário de Belo Horizonte (UniBH), Belo Horizonte, Brazil; ^5^Faculdade de Ciências Médicas de Minas Gerais, Belo Horizonte, Brazil; ^6^Pontifícia Universidade Católica de Minas Gerais, Betim, Brazil; ^7^Hospital Risoleta Tolentino Neves, Belo Horizonte, Brazil; ^8^Universidade Federal dos Vales do Jequitinhonha e Mucuri, Diamantina, Brazil; ^9^Hospital Universitário de Santa Maria/EBSERH, Santa Maria, Brazil; ^10^Department of Internal Medicine, Universidade Federal de Santa Maria, Santa Maria, Brazil; ^11^Hospitais da Rede Mater Dei, Belo Horizonte, Brazil; ^12^Hospital Julia Kubitschek, Belo Horizonte, Brazil; ^13^Hospital Santa Rosália, Teófilo Otoni, Brazil; ^14^Hospital João XXIII, Belo Horizonte, Brazil; ^15^Faculdade de Medicina de Botucatu, Universidade Estadual Paulista Júlio de Mesquita Filho, Botucatu, Brazil; ^16^Hospital Mãe de Deus, Porto Alegre, Brazil; ^17^Hospital Universitário de Canoas, Canoas, Brazil; ^18^Hospital Nossa Senhora da Conceição and Hospital Cristo Redentor, Porto Alegre, Brazil; ^19^Instituto Orizonti, Belo Horizonte, Brazil; ^20^Hospital Santo Antônio, Curvelo, Brazil; ^21^Hospital Santa Casa de Misericórdia de Belo Horizonte, Belo Horizonte, Brazil; ^22^Hospital Universitário Ciências Médicas, Belo Horizonte, Brazil; ^23^Institute for Health Technology Assessment (IATS), Porto Alegre, Brazil; ^24^Hospital Bruno Born, Lajeado, Brazil; ^25^Hospital Metropolitano Odilon Behrens, Belo Horizonte, Brazil; ^26^Hospital Santa Cruz, Santa Cruz do Sul, Brazil; ^27^Universidade Federal de São João Del Rei, Divinópolis, Brazil; ^28^Hospital Eduardo de Menezes, Belo Horizonte, Brazil; ^29^Hospital Semper, Belo Horizonte, Brazil; ^30^Telehealth Center, University Hospital, Universidade Federal de Minas Gerais, Belo Horizonte, Brazil

**Keywords:** SARS-CoV-2, COVID-19, intensive care unit, prognosis, mortality, risk scores

In the published article, there was an error in Table 2 as published. The *p*-value for the comparison between ABC_2_-SPH and SAPS-3 (in line 7 of the table) was displayed as 0.0046, but the correct p-value is 0.0446. The corrected Table 2 and its caption “Comparison between ABC_2_-SPH and other scores.” appear below. The authors apologize for this error and state that this does not change the scientific conclusions of the article in any way. The original article has been updated.

**Table 2:** Comparison between ABC_2_-SPH and other scores.

**Reference score**	**Compared score**	***p*-value**	alpha^*^	***n***	**Result**
ABC_2_-SPH	Altschul et al.	0.9346	0.0063	1,094	AUROC of ABC_2_-SPH is not different
ABC_2_-SPH	4C Mortality Score	0.8878	0.0063	815	AUROC of ABC_2_-SPH is not different
ABC_2_-SPH	CURB-65	**0.0010**	0.0063	1,823	AUROC of ABC_2_-SPH is larger^**^
ABC_2_-SPH	SOARS	**0.0000**	0.0063	2,147	AUROC of ABC_2_-SPH is larger^**^
ABC_2_-SPH	SOFA	**0.0032**	0.0063	842	AUROC of ABC_2_-SPH is larger^**^
ABC_2_-SPH	Modified CHA2DS2-VASC	**0.0000**	0.0063	2,380	AUROC of ABC_2_-SPH is larger^**^
ABC_2_-SPH	SAPS-3	0.0446	0.0063	539	AUROC of ABC_2_-SPH is not different
ABC_2_-SPH	NEWS2	**0.0000**	0.0063	976	AUROC of ABC_2_-SPH is larger^**^

AUROC, area under the ROC curve.

* ue to the multiple comparisons, alpha was corrected using the Bonferroni method.

** ABC_2_-SPH has higher discrimination ability.

The bold values indicate which *p*-values are lower than the alpha, meaning that the AUROC of ABC2-SPH is larger than that of the score being compared.

