# Supplementary material for: Assessment of risk scores to predict mortality of COVID-19 patients admitted to the intensive care unit
Source: Front Med (Lausanne). 2023 Apr 20;10:1130218. doi: 10.3389/fmed.2023.1130218 (PMC10157088; doi:10.3389/fmed.2023.1130218)
Supplement: Supplementary file 1 [file Table_1.docx]

| **Supplementary Table S1.** Demographic and clinical characteristics for derivation and validation cohorts of patients admitted to hospital with COVID-19. | | | | | | | |
| --- | --- | --- | --- | --- | --- | --- | --- |
| **Characteristics** | **Total  n=3037^1^** | **Non-missing cases (%)** | **Died  n=1518^1^** | **Non-missing cases (%)** | **Discharged alive  n= 1519^1^** | **Non-missing cases (%)** | **p-value^2^** |
| Age (years) | 61.0 [50.0; 70.0] | 3037 (100%) | 65.0 [56.0, 73.8] | 1518 (100%) | 57.0 [46.0, 66.0] | 1519 (100%) | < 0.001 |
| Sex at birth male | 1697 (55.9%) | 3037 (100%) | 869 (57.2%) | 1518 (100%) | 828 (54.5%) | 1519 (100%) | 0.138 |
| **Comorbidities** |  |  |  |  |  |  |  |
| Hypertension | 1766 (58.1%) | 3037 (100%) | 975 (64.2%) | 1518 (100%) | 791 (52.1%) | 1519 (100%) | < 0.001 |
| Coronary artery disease | 146 (4.8%) | 3037 (100%) | 94 (6.2%) | 1518 (100%) | 52 (3.4%) | 1519 (100%) | < 0.001 |
| Heart failure | 160 (5.3%) | 3037 (100%) | 101 (6.7%) | 1518 (100%) | 59 (3.9%) | 1519 (100%) | < 0.001 |
| Atrial fibrillation or flutter | 60 (2.0%) | 3037 (100%) | 35 (2.3%) | 1518 (100%) | 25 (1.6%) | 1519 (100%) | 0.191 |
| Stroke | 65 (2.1%) | 3037 (100%) | 40 (2.6%) | 1518 (100%) | 25 (1.6%) | 1519 (100%) | 0.060 |
| COPD | 114 (3.8%) | 3037 (100%) | 71 (4.7%) | 1518 (100%) | 43 (2.8%) | 1519 (100%) | 0.007 |
| Diabetes mellitus | 1001 (33.0%) | 3037 (100%) | 589 (38.8%) | 1518 (100%) | 412 (27.1%) | 1519 (100%) | < 0.001 |
| Obesity (BMI>30kg/m^2^) | 851 (28.0%) | 3037 (100%) | 428 (28.2%) | 1518 (100%) | 423 (27.8%) | 1519 (100%) | 0.831 |
| Cirrhosis | 6 (0.2%) | 3037 (100%) | 4 (0.3%) | 1518 (100%) | 2 (0.1%) | 1519 (100%) | 0.414 |
| Cancer | 65 (2.1%) | 3037 (100%) | 38 (2.5%) | 1518 (100%) | 27 (1.8%) | 1519 (100%) | 0.167 |
| Number of comorbidities |  | 3037 (100%) |  | 1518 (100%) |  | 1519 (100%) | < 0.001 |
| 0 | 828 (27.3%) |  | 341 (22.5%) |  | 487 (32.1%) |  |  |
| 1 | 917 (30.2%) |  | 431 (28.4%) |  | 486 (32.0%) |  |  |
| 2 | 703 (23.1%) |  | 390 (25.7%) |  | 313 (20.6%) |  |  |
| 3 | 473 (15.6%) |  | 276 (18.2%) |  | 197 (13.0%) |  |  |
| ≥ 4 | 116 (3.8%) |  | 80 (5.3%) |  | 36 (2.4%) |  |  |
| **Clinical assessment** |  |  |  |  |  |  |  |
| Heart rate (bpm) | 86.0 [75.0; 99.0] | 2956 (97%) | 88.0 [75.0, 101.0] | 1475 (97%) | 85.0 [75.0, 96.0] | 1481 (97%) | 0.001 |
| SF ratio | 101.1 [95.0; 160.0] | 2504 (82%) | 98.9 [93.7; 141.4] | 1248 (82%) | 105.3 [96.8; 192.0] | 1256 (83%) | < 0.001 |
| Respiratory rate (irpm) | 24.0 [20.0; 28.0] | 2535 (83%) | 24.0 [20.0; 28.0] | 1239 (82%) | 24.0 [20.0; 29.0] | 1296 (85%) | 0.110 |
| GCS < 15 | 190 (6.8%) | 2787 (92%) | 120 (8.7%) | 1379 (91%) | 70 (5.0%) | 1408 (93%) | < 0.001 |
| Systolic blood pressure |  | 2585 (85%) |  | 1283 (85%) |  | 1302 (86%) | < 0.001 |
| ≥ 90 mmHg | 1973 (76.3%) |  | 864 (67.3%) |  | 1109 (85.2%) |  |  |
| < 90 mmHg | 30 (1.2%) |  | 23 (1.8%) |  | 7 (0.5%) |  |  |
| Inotrope requirement | 582 (22.5%) |  | 396 (30.9%) |  | 186 (14.3%) |  |  |
| Diastolic blood pressure |  | 2560 (84%) |  | 1273 (84%) |  | 1287 (85%) | < 0.001 |
| > 60 mmHg | 1729 (67.5%) |  | 741 (58.2%) |  | 988 (76.8%) |  |  |
| ≤ 60 mmHg | 249 (9.7%) |  | 136 (10.7%) |  | 113 (8.8%) |  |  |
| Inotrope requirement | 582 (22.7%) |  | 396 (31.1%) |  | 186 (14.5%) |  |  |
| FiO_2_ (%) | 0.9 [0.6; 0.9] | 2527 (83%) | 0.9 [0.7; 1.0] | 1262 (83%) | 0.9 [0.5; 0.9] | 1265 (83%) | < 0.001 |
| O_2_ saturation (%) | 94.0 [90.0; 96.0] | 2994 (99%) | 93.0 [90.0; 96.0] | 1492 (98%) | 94.0 [91.2; 97.0] | 1502 (99%) | < 0.001 |
| **Laboratory parameters** |  |  |  |  |  |  |  |
| Platelet count (10^9^/L) | 229 [178; 294] | 2907 (96%) | 212 [165.5; 275.5] | 1443 (95%) | 244 [194; 308.25] | 1464 (96%) | < 0.001 |
| Creatinine (mg/dL) | 0.9 [0.7; 1.4] | 2616 (86%) | 1.1 [0.8; 1.9] | 1289 (85%) | 0.8 [0.7; 1.1] | 1327 (87%) | < 0.001 |
| Urea, mg/dL | 49.0 [34.1; 77.0] | 2852 (94%) | 60.0 [40.0; 94.7] | 1422 (94%) | 42.0 [30.0; 58.7] | 1430 (94%) | < 0.001 |
| C-reactive protein (mg/L) | 120.0 [71.8; 198.0] | 2219 (73%) | 132.7 [81.9; 216.8] | 1114 (73%) | 107.3 [62.0; 186.0] | 1105 (73%) | < 0.001 |
| pO_2_ (mmHg) | 77.0 [62.6; 99.5] | 2862 (94%) | 75.0 [61.0; 97.5] | 1430 (94%) | 78.0 [64.7; 102.6] | 1432 (94%) | < 0.001 |
| pCO_2_ (mmHg) | 39.0 [34.0; 37.0] | 2614 (86%) | 40.0 [34.0; 49.5] | 1292 (85%) | 38.0 [34.0; 44.3] | 1322 (87%) | < 0.001 |
| **Score calculation** |  |  |  |  |  |  |  |
| SOFA | 5 [3; 8] | 329 (11%) | 7 [4; 9] | 168 (11%) | 4 [3; 6] | 158 (10%) | < 0.001 |
| SAPS-3 | 54 [46; 62] | 541 (18%) | 55 [49; 63] | 305 (20%) | 51 [42; 59] | 236 (16%) | < 0.001 |
| *^1^*n (%); Median (IQR) *^2^*Pearson's Chi-squared test; Wilcoxon rank sum test; Fisher's exact test. BMI: body mass index; COPD: chronic obstructive pulmonary disease; GCS: Glasgow Coma Score; NLR: neutrophils-to-lymphocytes ratio; SF ratio: SpO_2_/FiO_2_ ratio. | | | | | | | |
