# Supplementary material for: Assessment of risk scores to predict mortality of COVID-19 patients admitted to the intensive care unit
Source: Front Med (Lausanne). 2023 Apr 20;10:1130218. doi: 10.3389/fmed.2023.1130218 (PMC10157088; doi:10.3389/fmed.2023.1130218)
Supplement: Supplementary file 2 [file Table_2.docx]

| **Supplementary Table S2.** Discrimination ability for each score applied in the database of COVID-19 patients admitted to the intensive care unit between March 1, 2021, and April 30, 2021. | | |
| --- | --- | --- |
| **Model** | **N*** | **AUROC (95%CI)** |
| ABC_2_-SPH | 1,210 | 0.725 (0.697-0.752) |
| Altschul et al | 1,009 | 0.722 (0.691-0.751) |
| 4C Mortality Score | 717 | 0.718 (0.682-0.754) |
| CURB-65 | 1,592 | 0.662 (0.636-0.687) |
| SOARS | 1,896 | 0.655 (0.631-0.679) |
| Modified CHA2DS2-VASc | 2,097 | 0.639 (0.616-0.663) |
| NEWS2 | 762 | 0.603 (0.560-0.643) |
| SOFA | 468 | 0.596 (0.546-0.644) |
| SAPS-3 | 269 | 0.537 (0.466-0.605) |
| *Complete case analysis. Data were imputed for variables with up to 30% missing values. | | |
