# Supplementary material for: Assessment of risk scores to predict mortality of COVID-19 patients admitted to the intensive care unit
Source: Front Med (Lausanne). 2023 Apr 20;10:1130218. doi: 10.3389/fmed.2023.1130218 (PMC10157088; doi:10.3389/fmed.2023.1130218)
Supplement: Supplementary file 3 [file Table_3.docx]

| **Supplementary Table S3.** Deaths according to age group. | | | |
| --- | --- | --- | --- |
| **Age group (years)** | **Died** | **N** | **Deaths/N (%)** |
| 18-29 | 21 | 82 | 25.6 |
| 30-39 | 65 | 225 | 28.9 |
| 40-49 | 143 | 427 | 33.5 |
| 50-59 | 299 | 675 | 44.3 |
| 60-69 | 431 | 797 | 54.1 |
| 70-79 | 370 | 570 | 64.9 |
| ≥ 80 | 189 | 261 | 72.4 |
